# Hemoglobinopathies and Thalassemia in Northeast India: A Scoping Review of Disease Burden, Mutation Spectrum, and Gaps in Molecular Genetic Evidence

**DOI:** 10.7759/cureus.115076

**Published:** 2026-08-24

**Authors:** Mauchumi S Pathak, Syed Javed S Chisty, Parismita Pathak, Monalisha S Borah, Tahmina Mazumder

**Affiliations:** 1 Department of Biochemistry, Gauhati Medical College and Hospital, Guwahati, IND; 2 Department of Computer Science, Indian Institute of Information Technology Guwahati, Guwahati, IND; 3 Multidisciplinary Research Unit, Gauhati Medical College and Hospital, Guwahati, IND; 4 Department of Computational Biology, Mazumdar Shaw Medical Foundation, Guwahati, IND

**Keywords:** genetic epidemiology, hemoglobin e, hemoglobinopathies, northeast india, prisma-scr, thalassemia

## Abstract

Hemoglobinopathies are among the most prevalent inherited monogenic disorders globally and represent a major public health challenge in India, which contributes substantially to the global disease burden. Marked regional heterogeneity exists across the country, and Northeast India constitutes a genetically distinct region characterized by a high prevalence of hemoglobin E (HbE), significant tribal population diversity, and a disproportionate burden of compound hemoglobin disorders. This scoping review aimed to systematically map the available evidence on the burden, geographic distribution, diagnostic patterns, and molecular spectrum of hemoglobinopathies globally and in India, with a primary analytical focus on Northeast India. A scoping review was undertaken in accordance with the Preferred Reporting Items for Systematic Reviews and Meta-Analyses extension for Scoping Reviews (PRISMA-ScR) standards. We conducted extensive literature searches in PubMed, Google Scholar, and Web of Science and supplemented these with citation tracking. We included primary studies providing epidemiological, hematological, or molecular data on hemoglobinopathies. Eligible studies were population- and hospital-based. Data on research setting, demographic characteristics, diagnostic procedures, distribution of hemoglobin variants, and mutation profiles were extracted and analyzed using descriptive and thematic synthesis. Forty-five studies were included. Of these, 27 were undertaken exclusively in Northeast India, 11 were pan-Indian or multi-regional studies that published stratified data for Northeast Indian populations, and seven provided global or Southeast Asian contextual evidence. HbE was the most common structural hemoglobin variant observed across all districts of Northeast India in heterozygous, homozygous, and compound heterozygous states. HbE-β-thalassemia was consistently recognized as a primary cause of clinically significant disease, whereas reports of HbS-β-thalassemia were less frequent and largely limited to isolated clinical and hospital-based reports. The spectrum of β-thalassemia mutations in Northeast India overlapped considerably with pan-Indian patterns, with recurrent mutations such as IVS-I-5 (G>C), IVS-I-1 (G>T), and codon 41/42 (-CTTT), in addition to rare and region-specific mutations. Hospital-based and molecular investigations suggest that compound hemoglobinopathies are underdiagnosed and underline the modifying influence of α-thalassemia and other hereditary factors. A number of studies also reported a disproportionate burden among tribal populations, reflecting founder effects, endogamy, and disparities in access to healthcare. Northeast India exhibits a distinct hemoglobinopathy landscape dominated by HbE and compound hemoglobin disorders, with unique molecular and clinical characteristics. These findings underscore the need for region-specific screening strategies, expanded molecular diagnostic panels, and targeted public health interventions tailored to the genetic and sociocultural context of Northeast India.

## Introduction and background

Hemoglobinopathies constitute one of the most common groups of inherited monogenic disorders worldwide, affecting millions of individuals and contributing substantially to childhood morbidity and mortality [1,2]. These disorders arise from mutations in globin genes that either alter the structure of hemoglobin molecules or reduce the synthesis of globin chains, leading to a broad spectrum of clinical phenotypes ranging from asymptomatic carrier states to severe, transfusion-dependent anemia.

Globally, β-thalassemia and sickle cell disease account for the majority of clinically significant hemoglobinopathies. Their high prevalence in specific geographic regions is closely linked to historical malaria endemicity, where heterozygous carrier states confer a selective survival advantage, resulting in positive selection and long-term persistence of these mutations in human populations [3]. Hemoglobin E (HbE), caused by a point mutation at codon 26 of the β-globin gene, is among the most prevalent structural hemoglobin variants worldwide and is particularly common in Southeast Asia and parts of the Indian subcontinent [4].

India bears a disproportionate share of the global hemoglobinopathy burden owing to its large population size, extensive ethnic diversity, and complex demographic history shaped by ancient migrations, endogamy, and selective evolutionary pressures [5,6]. The distribution of hemoglobinopathies across India is highly heterogeneous, with marked regional clustering of specific variants. β-Thalassemia and sickle cell disease predominate in western, central, and southern India, whereas HbE shows a striking concentration in eastern and northeastern regions of the country [7,8].

Northeast India occupies a unique geographic and genetic position at the interface of South Asia and Southeast Asia. The region is characterized by substantial ethnic heterogeneity, encompassing Indo-Aryan, Tibeto-Burman, and Austroasiatic populations, many of whom practice long-standing endogamy [9]. This population structure, combined with historical migration routes and relative geographic isolation, has contributed to a distinct hemoglobinopathy landscape. Early population genetic surveys reported unusually high carrier frequencies of HbE in Assam and adjoining states, with some groups exhibiting prevalence levels comparable to those observed in Southeast Asia [10-12]. Recent molecular studies from Assam have also identified rare and previously unreported β-thalassemia mutations, further underscoring the genetic heterogeneity of the region [13].

A defining feature of hemoglobinopathies in Northeast India is the frequent occurrence of HbE in compound heterozygous states with β-thalassemia, resulting in HbE-β-thalassemia. This disorder is now recognized as a major cause of clinically significant hemoglobinopathy in the region [14-16]. HbE-β-thalassemia is characterized by remarkable clinical heterogeneity, with affected individuals presenting along a continuum ranging from mild anemia to severe, transfusion-dependent disease. Disease severity is influenced by the specific β-thalassemia mutation, co-inherited genetic modifiers such as α-thalassemia and fetal hemoglobin levels, and environmental factors [17,18].

In addition to HbE and β-thalassemia, other hemoglobin variants including hemoglobin S (HbS), hemoglobin D (HbD), and α-thalassemia have been reported in Northeast India, particularly among tribal populations [19,20]. Although less prevalent than HbE-related disorders, these variants are clinically relevant owing to their potential to form compound heterozygous states. Case-based and hospital-based studies from Assam have documented less common compound heterozygous interactions such as HbS-β-thalassemia, along with rare and previously underreported β-thalassemia mutations, underscoring the molecular complexity of hemoglobinopathies in the region [13,21]. Hospital-based diagnostic studies from tertiary care centers in Assam have further characterized the regional spectrum of hemoglobin variants and β-thalassemia mutations, providing early evidence of compound hemoglobinopathies and mutation heterogeneity in Northeast India [22].

Despite decades of research, the evidence base on hemoglobinopathies in Northeast India remains fragmented. Available data are scattered across population surveys, hospital-based diagnostic studies, molecular investigations, and isolated case reports. Furthermore, several pan-Indian studies do not stratify data sufficiently for Northeast Indian groups, limiting their usefulness for this genetically unique region. Synthesis and comparison across studies are further complicated by variability in the diagnostic methodologies used, ranging from hematological indices and electrophoresis to high-performance liquid chromatography (HPLC) and molecular procedures.

Given this heterogeneity in populations, methods, and outcomes, a scoping review is particularly well suited to systematically map the existing literature, identify key epidemiological and molecular trends, and highlight knowledge gaps within the context of hemoglobinopathies in Northeast India. Rather than answering narrowly specified questions, a scoping review allows for the inclusion of different types of evidence and provides a framework for understanding a complex and evolving research landscape.

Thus, the objectives of this scoping review were to map the burden and distribution of hemoglobinopathies in Northeast India; review the spectrum of hemoglobin variants and thalassemia mutations reported in the region; synthesize evidence on compound hemoglobinopathies, particularly HbE-β-thalassemia and other clinically relevant compound hemoglobinopathies; and identify trends in methodology, gaps in knowledge, and priorities for future research and public health interventions.

## Review

Methods

Study Design and Methodological Framework

This study was conducted as a scoping review based on the Preferred Reporting Items for Systematic Reviews and Meta-Analyses extension for Scoping Reviews (PRISMA-ScR) guidelines. A scoping review methodology was preferred over a systematic review or meta-analysis because the evidence on hemoglobinopathies in Northeast India is highly heterogeneous, comprising population-based surveys, hospital-based observational studies, molecular genetic analyses, newborn screening reports, and case series conducted across different settings.

In line with scoping review methods, the main objective was to map the extent, nature, and thematic distribution of the existing evidence rather than to derive pooled prevalence estimates. Therefore, no formal risk-of-bias assessment or quantitative meta-analysis was performed.

Eligibility Criteria

Inclusion criteria: Studies were eligible if they reported primary data on hemoglobinopathies or thalassemia and included populations from one or more Northeast Indian states (Assam, Arunachal Pradesh, Manipur, Meghalaya, Mizoram, Nagaland, Tripura, or Sikkim), either exclusively or as clearly defined subgroups within multicentric or pan-Indian studies. Eligible studies provided epidemiological, hematological, molecular, or clinical data and used laboratory-based diagnostic methods such as HPLC, hemoglobin electrophoresis, or molecular genetic testing. Both population-based and hospital-based studies were included to capture the diversity of research conducted in the region. In addition, selected national, Southeast Asian, and global studies were included as contextual evidence when they provided comparative epidemiological, molecular, clinical, or public health insights relevant to interpreting findings from Northeast India.

Exclusion criteria: Studies were excluded if they lacked extractable data relevant to hemoglobinopathies in Northeast India, focused on non-hemoglobin genetic disorders, represented duplicate publications, or lacked sufficient methodological detail. Editorials, conference abstracts, letters to the editor, and commentaries were excluded. Review articles were not included as primary evidence sources; however, selected review articles and global or Southeast Asian studies were retained as contextual evidence when they provided important epidemiological, molecular, clinical, or historical perspectives relevant to interpreting findings from Northeast India.

Information Sources and Search Strategy

A comprehensive literature search was performed using the electronic databases PubMed, Web of Science, and Google Scholar, including all records available up to June 2025. To ensure comprehensive coverage, reference lists of included articles were also screened to identify additional relevant studies, and manual reference list screening (citation chasing) was performed for all included articles. Search strategies were developed using combinations of keywords and Boolean operators related to hemoglobinopathies and the Northeast Indian region, including "hemoglobinopathy", "thalassemia", "β-thalassemia", "α-thalassemia", "hemoglobin E", "HbE", "hemoglobin S", "mutation", "genetic", "Assam", "Tripura", "Manipur", "Arunachal Pradesh", and "Northeast India". Given substantial variability in terminology across older and contemporary studies, broad search strings were intentionally employed to maximize sensitivity.

The database-specific search strategies were adapted according to the indexing and search functions of each database. The PubMed search used Medical Subject Headings (MeSH) terms and Title/Abstract fields: ("Hemoglobinopathies"[MeSH Terms] OR hemoglobinopathy*[Title/Abstract] OR haemoglobinopathies*[Title/Abstract] OR thalassemia[Title/Abstract] OR thalassaemia[Title/Abstract] OR "β-thalassemia"[Title/Abstract] OR "α-thalassemia"[Title/Abstract] OR "hemoglobin E"[Title/Abstract] OR HbE[Title/Abstract] OR "hemoglobin S"[Title/Abstract] OR "hemoglobin variant*"[Title/Abstract] OR mutation*[Title/Abstract] OR genetic*[Title/Abstract]) AND ("Northeast India"[Title/Abstract] OR "North East India"[Title/Abstract] OR Assam[Title/Abstract] OR Tripura[Title/Abstract] OR Manipur[Title/Abstract] OR "Arunachal Pradesh"[Title/Abstract] OR Meghalaya[Title/Abstract] OR Mizoram[Title/Abstract] OR Nagaland[Title/Abstract] OR Sikkim[Title/Abstract]).

The Google Scholar search used the corresponding keyword and phrase combinations, and the search strings were as follows: ("hemoglobinopathy*" OR "haemoglobinopathies*" OR thalassemia OR thalassaemia OR "β-thalassemia" OR "α-thalassemia" OR "hemoglobin E" OR HbE OR "hemoglobin S" OR "hemoglobin variant*" OR mutation* OR genetic*) AND ("Northeast India" OR "North East India" OR Assam OR Tripura OR Manipur OR "Arunachal Pradesh" OR Meghalaya OR Mizoram OR Nagaland OR Sikkim).

The Web of Science search used the Topic (TS) field: TS=(hemoglobinopathy OR haemoglobinopathies OR thalassemia OR thalassaemia OR "β-thalassemia" OR "α-thalassemia" OR "hemoglobin E" OR HbE OR "hemoglobin S" OR "hemoglobin variant*" OR mutation* OR genetic*) AND TS=("Northeast India" OR "North East India" OR Assam OR Tripura OR Manipur OR "Arunachal Pradesh" OR Meghalaya OR Mizoram OR Nagaland OR Sikkim). These searches were supplemented by manual screening of the reference lists of relevant articles and reviews to identify additional potentially eligible studies. The complete search strategy is provided in the Appendices.

Study Selection Process

All records retrieved through database searches were imported into a reference management system, and duplicate entries were removed. Titles and abstracts were screened for relevance against the predefined eligibility criteria, and articles considered potentially eligible were subjected to full-text assessment.

Disagreements during screening or full-text review were resolved through iterative review and consensus. The study selection process is summarized in the PRISMA-ScR flow diagram. Three additional studies flagged by automated screening tools were manually reviewed at the recommendation of an expert and were subsequently included after meeting all eligibility criteria.

Data Extraction and Charting

Data extraction strategy: Data extraction was conducted using a predefined data-charting framework, developed iteratively to align with the review objectives and the nature of the available literature. Each included study was reviewed in full, and data were extracted manually. Extracted variables included bibliographic details (authors and year of publication), study design, geographic location within Northeast India, characteristics of the study population, diagnostic methods used, hemoglobin variants identified, β-thalassemia mutation profiles, and relevant clinical features such as disease severity and transfusion requirements.

Handling heterogeneity across studies: Extracted data were not pooled quantitatively owing to substantial heterogeneity in study designs, populations, diagnostic approaches, and outcome reporting. Findings were instead synthesized using a narrative and thematic approach. Evidence was interpreted according to geographic distribution, population characteristics (tribal versus non-tribal), types of hemoglobinopathies reported, and diagnostic methods used. This approach allowed for meaningful synthesis while avoiding inappropriate comparisons across fundamentally different types of study.

Data Synthesis

Extracted data were descriptively and thematically synthesized according to scoping review methodology. Results were clustered into the following thematic areas: overall burden and distribution of hemoglobinopathies; major hemoglobin variants in Northeast India; spectrum of β-thalassemia mutations; evidence on compound hemoglobinopathies, particularly HbE-β-thalassemia and other clinically relevant compound hemoglobinopathies; descriptive patterns reported in studies focusing on tribal and non-tribal populations; and temporal trends in diagnostic practices.

Results are presented using narrative synthesis supported by summary tables, facilitating structured comparison across studies while preserving contextual nuance.

PRISMA-ScR Statement

This manuscript was prepared in accordance with the PRISMA-ScR checklist, and all recommended reporting items have been addressed.

Ethical Considerations

As this study involved the synthesis of previously published data, ethical approval was not required. All information was obtained from publicly available sources, and appropriate citation of original studies was ensured throughout.

Results

Study Selection and Overview

The systematic database search and manual reference screening yielded a substantial body of literature addressing hemoglobinopathies in India. Following the removal of duplicates and the application of the eligibility criteria, 45 studies were included in this scoping review. Of these, 27 were conducted exclusively in Northeast India, 11 were pan-Indian or multi-regional studies containing Northeast-specific data, and seven were contextual global or Southeast Asian studies included to provide comparative perspectives on disease epidemiology, mutation spectra, and clinical manifestations.

The included studies spanned more than five decades, reflecting the evolution of hemoglobinopathy research in Northeast India from early population genetic surveys to contemporary molecular and clinical investigations [9,10,15,23].

Study designs included population-based surveys, hospital-based observational studies, newborn screening programs, molecular genetic analyses, and clinically informative case reports. Assam contributed the largest number of studies, followed by Tripura and Manipur, with fewer studies from Arunachal Pradesh, Meghalaya, Mizoram, and Nagaland [19,24].

**Figure 1:**
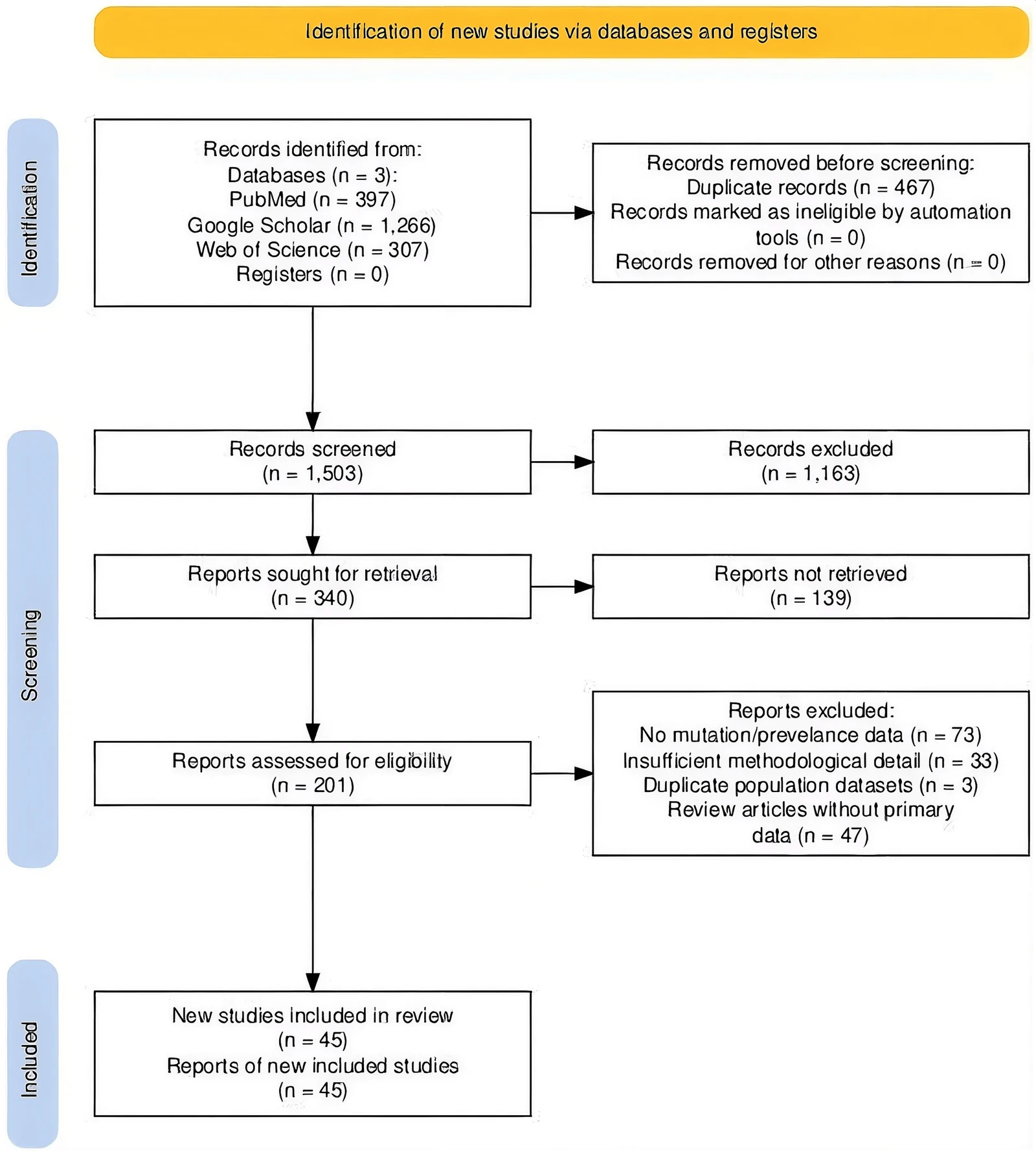
PRISMA 2020 flow diagram illustrating the identification, screening, eligibility assessment, and inclusion of studies. Three studies initially excluded by automated screening were manually reviewed and included following expert recommendation PRISMA: Preferred Reporting Items for Systematic Reviews and Meta-Analyses

The study selection process is summarized in the PRISMA-ScR flow diagram (Figure 1).

Characteristics of the Included Studies

The included studies demonstrated marked heterogeneity in study design, population characteristics, and diagnostic methodology. Population-based surveys primarily focused on estimating carrier frequencies and variant distribution among tribal and non-tribal populations [11,12,25]. Hospital-based studies provided detailed insights into clinical severity, transfusion requirements, and compound heterozygosity, particularly in pediatric cohorts [15,16,22,26].

Molecular genetic studies played a critical role in identifying the β-thalassemia mutation spectrum and rare variants specific to the Indian subcontinent [27-29]. Over time, there was a clear methodological shift from hematological indices and electrophoresis toward HPLC and molecular diagnostics.

The geographic scope of the included studies is summarized in Table 1.

**Table 1:** Geographic scope of the included studies

Geographic scope	Number of studies	Description
Northeast India only	27	Assam, Tripura, Manipur, Arunachal Pradesh
Pan-Indian/multi-regional	11	Included Northeast populations
Global/Southeast Asia	7	Contextual and comparative

Classification of the included evidence: The included evidence comprised three categories: (i) Northeast India-specific primary studies; (ii) pan-Indian or multi-regional studies reporting Northeast-specific findings; and (iii) contextual global and Southeast Asian studies. Contextual studies were analyzed separately from the primary evidence base and were used to support the interpretation of regional epidemiological and molecular trends.

Geographic Distribution of Hemoglobinopathy Studies in Northeast India

Most of the included studies were from Assam, likely reflecting its large population size and the availability of tertiary care and research institutions. Multiple studies reported a high prevalence of HbE, β-thalassemia trait, and compound HbE-β-thalassemia from Assam [10,15,26].

Tripura also emerged as an important region, with population-based surveys and newborn screening activities revealing the burden of HbE and β-thalassemia among both tribal and non-tribal populations. Studies from Manipur reported that the prevalence of hemoglobinopathies varies between migrant and indigenous populations, reflecting population admixture [25]. In contrast, Arunachal Pradesh, Meghalaya, Mizoram, and Nagaland were underrepresented in the literature, with available evidence largely limited to small population surveys or comparative tribal studies [19,30].

The geographic distribution and study characteristics of the included studies are summarized in Table 2.

**Table 2:** Geographic scope and study characteristics of the included studies in Northeast India HbE: hemoglobin E; HbS: hemoglobin S

State/region	Approximate study representation	Dominant study type	Key focus
Assam	High	Hospital- and population-based	HbE, β-thalassemia, HbE-β-thalassemia
Tripura	Moderate	Population surveys, newborn screening	HbE, β-thalassemia
Manipur	Moderate	Population-based	HbE, β-thalassemia
Arunachal Pradesh	Low	Tribal surveys	HbE, HbS
Meghalaya, Mizoram, Nagaland	Very low	Small surveys/case series	Limited data

Burden of Hemoglobinopathies: Global, Indian, and Northeast Indian Context

Across the included literature, hemoglobinopathies were consistently recognized as a major inherited disease burden at the global and national levels [1,2]. While β-thalassemia and sickle cell disease dominate the global and pan-Indian burden, Northeast India demonstrated a distinct hemoglobinopathy profile characterized by the predominance of HbE and compound hemoglobinopathies [5,8].

Population-based surveys from Northeast India repeatedly identified HbE as the most prevalent hemoglobin variant, often exceeding the frequencies of β-thalassemia trait reported in other regions of India [10-12]. Hospital-based studies further indicated that HbE-β-thalassemia constituted a substantial proportion of clinically significant hemoglobinopathy cases presenting to tertiary care centers [14,15].

The burden of hemoglobinopathies by region is summarized in Table 3.

**Table 3:** Burden of hemoglobinopathies by region HbE: hemoglobin E; HbS: hemoglobin S

Region	Dominant variants	Key observations
Global	β-Thalassemia, HbS	Major inherited disease burden
India	β-Thalassemia, HbS, HbE	Strong regional heterogeneity
Northeast India	HbE, HbE-β-thalassemia (occasional HbS-related compounds)	Very high carrier frequencies

Spectrum of Hemoglobin Variants in Northeast India

Among the hemoglobin variants reported in Northeast India, structural hemoglobin variants were most common, with HbE being the most prevalent. HbE was consistently identified in heterozygous, homozygous, and compound heterozygous states across several population groups and geographic settings in the region [8-11]. The high population frequency of HbE underlies its major role in determining the regional burden of hemoglobinopathies.

HbS was reported less frequently and appeared largely confined to certain tribal groups and specific geographic areas, where it often co-existed with β-thalassemia or HbE, leading to compound hemoglobinopathies of variable clinical severity [19,28]. HbD has been reported very rarely in Northeast India but is of clinical significance, having been described to interact with β-thalassemia in isolated cases [26].

Evidence on α-thalassemia remains relatively limited compared with other variants. Nevertheless, several studies identified α-thalassemia as an important genetic modifier of the clinical expression of compound hemoglobinopathies, especially HbE-β-thalassemia [30].

Table 4 summarizes the relative frequencies, distribution patterns, and clinical relevance of the major hemoglobin variants described in Northeast India.

**Table 4:** Hemoglobin variants reported in Northeast India Relative frequency based on reported literature. HbE: hemoglobin E; HbS: hemoglobin S; HbD: hemoglobin D

Variant	Frequency pattern	Clinical relevance
HbE	Very common	Mild alone; severe in compound states
β-Thalassemia	Common	Major cause of transfusion-dependent anemia
HbE-β-thalassemia	Frequent	Wide clinical spectrum
HbS	Occasional	Mainly in tribal populations
HbD	Rare	Limited reports

Besides HbE-β-thalassemia, compound heterozygosity of HbS-β-thalassemia has also been reported in Northeast India, but appears rare and largely restricted to isolated clinical reports [13].

β-Thalassemia Mutation Spectrum

Molecular studies have shown considerable heterogeneity in the β-thalassemia mutation spectrum across Northeast India. Several mutations frequently reported across the Indian subcontinent were also consistently identified in cohorts from the region. Of these, IVS-I-5 (G>C) and codon 41/42 (-CTTT) were the most frequently reported, consistent with their established predominance in the Indian β-thalassemia mutation pool [27,28,31].

Beyond these common variants, a number of studies reported less frequent, region-specific splice-site and frameshift mutations, often detected in smaller or population-specific cohorts. The identification of these rare variants highlights the genetic diversity of Northeast India and the limitations of restricted mutation panels optimized for other parts of India [32,33]. Recent evidence from Assam, including the identification of rare β-thalassemia mutations such as -90 (C>T) and IVS-I-130 (G>C), has further expanded the regional mutation spectrum, pointing to the ongoing discovery of region-specific variants in Northeast India [13]. Large deletions, such as the 619 bp deletion, were reported only sporadically in Northeast India and appeared less prevalent than in certain other Indian regions where such deletions are more common [28].

A comparative overview of the major β-thalassemia mutations reported globally, within India, and in Northeast India, along with their mutation types and relative frequencies, is presented in Table 5.

**Table 5:** Common β-thalassemia mutations reported globally, in India, and in Northeast India Relative frequency based on reported literature.

Mutation	Mutation type	Global	India
IVS-I-5 (G>C)	Splice site	Common	Very common
IVS-I-1 (G>T)	Splice site	Common	Common
Codon 41/42 (-CTTT)	Frameshift	Global	Common
Codon 8/9 (+G)	Frameshift	Global	Common
619 bp deletion	Large deletion	Region-specific	Common in some regions

Tribal and Non-tribal Populations: Descriptive Pat

Several of the included studies specifically focused on tribal populations of Northeast India, reporting high carrier frequencies of hemoglobin variants, especially HbE, and distinct hemoglobinopathy profiles shaped by founder effects, genetic drift, and long-standing endogamy [5,19,20]. These studies provide valuable information on the burden and diversity of hemoglobinopathies within tribal populations.

In contrast, mixed or predominantly non-tribal cohorts, especially hospital cohorts from metropolitan and semi-urban areas, showed greater heterogeneity in hemoglobin variants, indicative of population admixture and migration patterns [15,25]. However, few studies directly compared tribal and non-tribal groups within the same study, and differences in study design, sampling strategy, and diagnostic approach limited quantitative comparisons of prevalence or risk.

Thus, the present data do not allow for conclusive comparisons of burden or risk between tribal and non-tribal communities but do point to descriptive differences in reported patterns of hemoglobinopathy across these populations.

Compound Hemoglobinopathies and Clinical Heterogeneity

Compound hemoglobinopathies, particularly HbE-β-thalassemia, emerged as the dominant clinically significant disorder across Northeast India. Hospital-based studies consistently reported wide inter-individual variability in disease severity, even among patients sharing similar genotypes [14,16].

Clinical heterogeneity was influenced by the specific β-thalassemia mutation, fetal hemoglobin levels, co-inheritance of α-thalassemia, and nutritional and environmental factors [17,18].

Evidence on α-Thalassemia and Modifying Effects

Although fewer studies have explicitly examined α-thalassemia, the available data suggest that its co-inheritance with HbE or β-thalassemia modifies clinical severity by affecting globin chain balance [30,34]. The limited molecular characterization of α-thalassemia remains an important research gap in Northeast India.

Diagnostic Approaches Across Studies

Earlier investigations relied mainly on hematological indices and electrophoresis for diagnosis [9,10]. Over time, HPLC became the most widely used diagnostic tool in hospital-based settings [15,35]. In select studies, molecular diagnostic techniques were used to confirm genotypes and define mutation spectra [27,36].

In conclusion, these findings reveal that Northeast India exhibits a unique and complex pattern of hemoglobinopathies, driven mainly by HbE and compound HbE-β-thalassemia, with substantial geographic, population, and molecular variation.

Discussion

This scoping review provides a detailed synopsis of the epidemiological, molecular, and clinical landscape of hemoglobinopathies in Northeast India, a region that presents a distinct genetic and public health profile within the Indian subcontinent. The reviewed evidence shows that Northeast India differs markedly from other regions of India in its predominant hemoglobin variants, mutation spectra, and clinical phenotypes, with HbE and compound hemoglobinopathies, particularly HbE-β-thalassemia and, less commonly, HbS-β-thalassemia, being the main contributors to disease burden.

A consistent and striking finding across the literature is that Northeast India represents a distinct hemoglobinopathy corridor rather than a simple extension of the broader Indian genetic landscape. Early population genetic studies documented an unusually high occurrence of HbE in Assam and adjoining areas, forming the basis for subsequent research [10,11,37]. These observations were later corroborated by studies showing increased HbE frequencies across several Northeast Indian states [8,12]. The prevalence of HbE in this region is comparable to that observed in Southeast Asia, particularly in Thailand, Cambodia, Laos, and Myanmar, where HbE is the most common structural hemoglobin variant [38,39]. Molecular and haplotype studies have revealed multiple origins of the β(E)-globin gene in Southeast Asia, supporting the hypothesis of ancient genetic continuity between Northeast India and these populations [40]. The geographic position of Northeast India, combined with historical migration routes, population admixture, and prolonged endogamy, has likely facilitated both the introduction and amplification of HbE. These evolutionary and demographic processes differentiate Northeast India from much of the Indian subcontinent, where β-thalassemia and sickle cell disease predominate [5,6].

Across the included studies, HbE emerged as the most prevalent hemoglobin variant in Northeast India, reported in heterozygous, homozygous, and compound heterozygous states [8-11]. While HbE trait and homozygous HbE disease are often clinically mild, the high population frequency of HbE substantially increases the likelihood of compound heterozygous states when co-inherited with β-thalassemia. HbE-β-thalassemia has consistently been identified as a major cause of clinically significant hemoglobinopathy in the region [14,15,26]. Unlike classical β-thalassemia major, HbE-β-thalassemia exhibits remarkable clinical heterogeneity, with patients presenting along a spectrum ranging from mild anemia to severe transfusion-dependent disease [17,41]. This heterogeneity complicates clinical management, prognostication, and genetic counseling. Several studies emphasize that disease severity in HbE-β-thalassemia cannot be reliably predicted from genotype alone [14,16], but instead reflects a complex interplay of genetic, environmental, and possibly epigenetic factors.

The molecular spectrum of β-thalassemia mutations reported from Northeast India includes both common Indian mutations and region-specific variants. Commonly identified mutations [27,28,31] included IVS-I-5 (G>C), IVS-I-1 (G>T), codon 41/42 (-CTTT), and codon 8/9 (+G). At the same time, several studies reported rare and novel mutations, highlighting the considerable genetic heterogeneity of the region [23,32,33]. This diversity has significant implications for diagnostic strategy, as reliance on limited mutation panels optimized for other regions of India may result in incomplete genetic characterization or missed diagnoses in Northeast India. The findings of this review therefore support the need for region-specific mutation panels and increased molecular diagnostic capacity tailored to the unique population structure of the region.

Although fewer studies have explicitly focused on α-thalassemia, available evidence suggests that its co-inheritance plays a significant modifying role in hemoglobinopathy phenotypes in Northeast India [30]. α-Thalassemia can ameliorate the globin chain imbalance characteristic of β-thalassemia and HbE-β-thalassemia by reducing excess α-globin chains, thereby influencing disease severity [34]. In addition to α-thalassemia, other genetic modifiers such as elevated fetal hemoglobin levels have been associated with reduced disease severity in HbE-β-thalassemia [7,17,18]. However, systematic exploration of these modifiers remains limited in Northeast India, representing an important gap in the existing evidence base.

The literature reviewed [5,19,20] consistently indicates that the burden of hemoglobinopathies is unevenly distributed among tribal populations of Northeast India. These populations often show high carrier frequencies of HbE and distinct hemoglobin variant profiles shaped by founder effects, genetic drift, and long-standing endogamy. Beyond genetic factors, tribal groups are often exposed to compounding layers of vulnerability arising from geographic remoteness, limited healthcare access, and socioeconomic disadvantage. These structural inequities exacerbate the clinical burden of hemoglobinopathies and constrain the effectiveness of current screening and treatment strategies, underscoring the importance of community-specific screening approaches and culturally competent genetic counseling programs.

The diagnostic modalities used across the included studies varied considerably over time. Earlier studies relied mainly on hematological markers and electrophoretic methods [9,37,42]. Although useful, these approaches lacked the precision to identify complex genotypes and rare variants. The advent of HPLC represented a significant advance, enabling more precise identification of HbE, HbS, and other variants [15,29,35]. However, HPLC alone is insufficient to fully characterize thalassemia mutations or identify genetic modifiers. Molecular diagnostic tools such as PCR-based assays and sequencing have enabled precise mutation detection and genotype-phenotype correlation [27,36]. Compound and atypical hemoglobinopathies in Northeast India are likely underdiagnosed and misclassified because of limited availability of molecular testing.

Northeast India is characterized by the predominance of HbE and a relatively higher incidence of HbE-β-thalassemia compared with other parts of India. Western and central India, by contrast, show a significant prevalence of β-thalassemia and sickle cell disease, with distinct mutation spectra and clinical characteristics [5,6,43-45]. Notably, the pattern of hemoglobinopathies in Northeast India more closely resembles that of Southeast Asia than that of much of India, not only in the prevalence of HbE but also in patterns of compound heterozygosity and clinical heterogeneity [8,38]. These similarities further support the concept of Northeast India as a genetic transition zone between South and Southeast Asia.

The findings of this scoping review carry important implications for public health. Traditional screening programs in India have focused predominantly on β-thalassemia and sickle cell disease, which may not be well suited to Northeast India, where HbE and compound hemoglobinopathies are more prevalent. Failure to account for this regional variation may result in the underestimation of disease burden and misdirected prevention strategies. Recent newborn screening initiatives in Tripura [24] demonstrate the feasibility and benefits of early detection in the Northeast Indian context. Scaling up such programs across the region, together with region-specific diagnostic algorithms and genetic counseling services, could substantially reduce disease burden and improve long-term outcomes.

While this review provides a substantial body of evidence, it also highlights important gaps in the existing research. Research output is unevenly distributed, with some states, particularly Assam, overrepresented and others underrepresented. Many studies are hospital-based and therefore not generalizable, and longitudinal data examining genotype-phenotype relationships remain scarce. Future studies should prioritize population-based surveys in understudied states, comprehensive molecular characterization of both α- and β-thalassemia, longitudinal clinical studies, and evaluation of culturally adapted screening and counseling approaches. In conclusion, the evidence collated in this review positions Northeast India as a hemoglobinopathy hotspot with distinct genetic, clinical, and public health characteristics. Addressing this burden will require diagnostic, therapeutic, and policy interventions tailored specifically to the region's hemoglobinopathy landscape.

Limitations

This scoping review relied solely on published literature and did not include unpublished data or grey literature. The considerable variability in study design, populations, diagnostic methods, and reporting style precluded quantitative synthesis and direct comparison across studies. Furthermore, literature on several Northeast Indian states remains underrepresented, and many included studies were hospital-based, which may limit generalizability. Nonetheless, the scoping review methodology enabled the extensive mapping of the existing literature and the identification of critical research gaps. The inclusion of selected contextual studies may introduce interpretive bias; however, these studies were analyzed separately from the primary evidence base and were used solely to contextualize regional findings.

## Conclusions

This PRISMA-ScR-guided scoping review provides an in-depth synthesis of the epidemiological, molecular, and clinical landscape of hemoglobinopathies in Northeast India, a region that constitutes a distinct and underrecognized genetic corridor within the Indian subcontinent. The evidence reviewed shows that Northeast India differs from most of India in its dominant hemoglobin variants, mutation spectra, and clinical phenotypes, with HbE and compound hemoglobinopathies, particularly HbE-β-thalassemia and, less frequently, HbS-β-thalassemia, being the principal drivers of disease burden. The variation in disease severity across studies highlights the inadequacy of prognosis based solely on genotype and underscores the importance of modifying factors, including the specific β-thalassemia mutation, fetal hemoglobin levels, and co-inheritance of α-thalassemia. This heterogeneity reflects the diverse population history of the region and carries important implications for diagnostic strategy. The findings of this review therefore support the need for region-specific mutation panels and greater access to genetic diagnostics.

The hemoglobinopathy profile of Northeast India more closely resembles that of Southeast Asia than that of other parts of India, further supporting its characterization as a transition zone between South and Southeast Asia, a finding with important implications for both research and policy. In conclusion, Northeast India is a hemoglobinopathy hotspot with a unique and complex genetic architecture that requires region-specific diagnostic, screening, and policy approaches. Strengthening population-based screening programs, expanding newborn screening, increasing molecular diagnostic capacity, and integrating culturally appropriate genetic counseling represent important steps toward reducing the burden of hemoglobinopathies and improving long-term outcomes in this region.

## Appendices

**Table 6:** Summary of the included studies (n=45) HbE: hemoglobin E; HbS: hemoglobin S; HPLC: high-performance liquid chromatography

Sl. no.	Author (year)	Study type	Geographic focus	Population	Key focus/contribution
1	Weatherall (2001) [3]	Narrative review	Global	General	Genotype-phenotype relationships in thalassemia
2	Weatherall (2010) [1]	Review	Global	General	Global burden of hemoglobinopathies
3	Williams and Weatherall (2012) [2]	Review	Global	General	Population genetics and global burden
4	Fucharoen and Winichagoon (2000) [4]	Review	Southeast Asia	Clinical cohorts	HbE-β-thalassemia clinical heterogeneity
5	Balgir (2010) [5]	Population genetics	Eastern India	Community	Genetic diversity of hemoglobinopathies
6	Verma et al. (2011) [6]	Review	India	Mixed	Thalassemia control in India
7	Bandyopadhyay et al. (1999) [7]	Molecular	Eastern India	Mixed	β-Globin mutation spectrum
8	Sikdar (2016) [8]	Review	Northeast India	Mixed	HbE origin, migration, impact
9	Das et al. (1971) [10]	Population survey	Assam	Community	Early HbE prevalence
10	Das et al. (1975) [37]	Population genetics	Assam	Mongoloid populations	HbE predominance
11	Nadkarni et al. (2019) [23]	Population survey	Assam, India	Community	HbE, G6PD, ABO
12	Deka (1981) [43]	Population study	Assam	Community	Hb genotype and fertility
13	Deka et al. (1987) [9]	Population survey	Northeast India	Mixed	Regional hemoglobinopathies
14	Deka et al. (1988) [11]	Population genetics	Assam	Endogamous groups	HbE distribution
15	Saha (1990) [12]	Population survey	Northeast India	Mongoloid groups	HbE frequency
16	Sengupta et al. (2002) [19]	Comparative survey	Arunachal, Tripura	Tribal	Tribal hemoglobinopathy patterns
17	Edison et al. (2008) [29]	Molecular study	India	General	Characterization of β-globin mutations and regional mutation heterogeneity
18	Fucharoen and Winichagoon (1997) [38]	Review	Southeast Asia, including Eastern/Northeastern Indian context	General	Molecular biology, population distribution, and clinical aspects of hemoglobinopathies in Southeast Asia
19	Achoubi et al. (2012) [25]	Population study	Manipur	Migrants	HbE and β-thalassemia prevalence
20	Baruah et al. (2014) [15]	Hospital-based	Assam	Mixed	HPLC profile (n≈9000)
21	Fucharoen and Winichagoon (2011) [39]	Review	Southeast Asia	General	Epidemiology, molecular basis, and clinical spectrum of β-thalassemia, HbE
22	Teli et al. (2016) [26]	Hospital-based	Assam	Mixed	HbE, HbS, β-thalassemia coexistence
23	Krishnamurti (2000) [14]	Clinical analysis	Northeast India	Pediatric	HbE-β-thalassemia underdiagnosis
24	Baruah and Baruah (2020) [16]	Hospital-based	Assam	Pediatric	HbE-β-thalassemia phenotypes
25	Cao et al. (1994) [17]	Review	Global	General	Genotype-phenotype modifiers
26	Kazazian et al. (1984) [27]	Molecular	India	Mixed	Early β-thalassemia mutation mapping
27	Colah et al. (2009) [28]	Multicentric molecular	India (including Northeast)	Mixed	Regional mutation heterogeneity
28	Varawalla et al. (1991) [32]	Molecular	India	Mixed	Rare β-thalassemia mutations
29	Nadkarni et al. (2009) [33]	Molecular	India	Mixed	Novel β-thalassemia mutations
30	Dastidar and Gajra (2013) [30]	Molecular	Assam, Arunachal	Tribal	α-Thalassemia and HbE interaction
31	Higgs (2013) [34]	Review	Global	General	Molecular basis of α-thalassemia
32	Colah et al. (2015) [20]	Review	India	Tribal	Sickle cell disease
33	Antonarakis et al. (1982) [40]	Molecular	Southeast Asia	General	Molecular analysis of the βE-globin gene
34	Upadhye et al. (2018) [24]	Newborn screening	Tripura	Newborns	Early detection feasibility
35	Sachdev et al. (2010) [35]	Hospital-based	India	Mixed	HPLC diagnostic utility
36	Vrettou et al. (2003) [36]	Methodological	Global	Carriers	PCR mutation screening
37	Thein et al. (1988) [18]	Molecular	Indian	Carriers	Genotype-phenotype relationships, β-globin mutation, prenatal diagnosis
38	Rees (2000) [41]	Review	Global	General	HbE/β-thalassemia
39	Madan et al. (2010) [44]	Population survey	India	Mixed	β-Thalassemia trait frequency
40	Panigrahi and Marwaha (2007) [42]	Review	India	Mixed	Mutation spectrum
41	Verma et al. (1997) [45]	Population-based genetic study	India	Carriers	Regional distribution and frequency of β-thalassemia mutations
42	Kumar et al. (2013) [31]	Molecular review	India	Mixed	Genetic heterogeneity
43	Pathak et al. (2014) [21]	Case report	Assam	Individual	HbS-β-thalassemia interaction
44	Borah et al. (2016) [22]	Hospital-based	Northeast India	Mixed	Hb variants and mutation patterns
45	Pathak and Borah (2023) [13]	Case report (molecular)	Assam	Individual	Rare β-thalassemia mutations
